# Breast cancer in women under 35 years

**DOI:** 10.1186/bcr3803

**Published:** 2015-11-05

**Authors:** Kate Hunter, Deirdre Boyle, John Kavanagh, Yvonne Hanhauser, Elizabeth Connolly, Sylvia O'Keeffe

**Affiliations:** 1 St James's Hospital, Dublin, Ireland

## Introduction

Breast cancer is rare in young women under 35 years; however, it can present a diagnostic challenge. This study was undertaken to determine the presentation of breast cancer in young women and the role of imaging including the predictive value in determining tumour size.

## Methods

All breast cancer diagnoses in women aged ≤35 years from 2006 to 2014 were identified. Data were then extracted from PACS and EPR. Analysis was performed on Microsoft Excel. Paired *t *tests were used to assess the accuracy of imaging in predicting final pathological tumour size.

## Results

Seventy patients with 74 presentations of carcinoma were included. Mean age 31 years (SD = 3.7). Of 73 examination scores (E): 7 % (5/73) were screen detected, 52 % (38/73) were E2−3 and 38 % (28/73) were suspected (E4−5). At ultrasound, 16 % (12/74) were U3 and 82 % (61/74) were suspected to be malignant (U4−5). Seventy-four per cent (51/69) had a mammogram score M4−M5. Seventy-five per cent (50/67) of patients were ACR density of 3−4. At MRI (42/70), tumour size correlated with final tumour size on pathology (*N *= 24, Pearson *R *0.45). There was no significant difference between MR estimates of size and final tumour size (*t *= −0.88, *p *= 0.39). In contrast, there was a significant difference between US size estimates and final pathology (*N *= 43, *t *= −2.56, *p* <0.05).

## Conclusion

Clinical examination has a low PPV in young women with ultrasound demonstrating a superior performance. However, 16 % of cancers were unsuspected at ultrasound. An important finding is the usefulness of MR in defining tumour size, suggesting it should be performed in all young patients.

